# Effective Construction of a High-Capacity Boronic Acid Layer on a Quartz Crystal Microbalance Chip for High-Density Antibody Immobilization

**DOI:** 10.3390/s19010028

**Published:** 2018-12-21

**Authors:** Pei-Heng Lin, Sheng-Cih Huang, Kuang-Po Chen, Bor-Ran Li, Yaw-Kuen Li

**Affiliations:** 1 Institute of Biomedical Engineering, College of Electrical and Computer Engineering, National Chiao Tung University, Hsinchu 30010, Taiwan; pamela.eed06g@nctu.edu.tw; 2 Department of Electrical and Computer Engineering, College of Electrical and Computer Engineering, National Chiao Tung University, Hsinchu 30010, Taiwan; 3 Department of Applied Chemistry, College of Science, National Chiao Tung University, Hsinchu 30010, Taiwan; Shengcih.bt96g@nctu.edu.tw (S.-C.H.); guangbo@g2.nctu.edu.tw (K.-P.C.); 4 Center for Emergent Functional Matter Science, National Chiao Tung University, Hsinchu 30010, Taiwan

**Keywords:** boronic acid, electrodeposition, quartz crystal microbalance, orientated antibody immobilization

## Abstract

Boronic acids (BAs) provide strong potential in orientation immobilization of antibody and the modification method is crucial for efficiency optimization. A highly effective method has been developed for rapid antibody immobilization on gold electrodes through the electrodeposition of a BA–containing linker in this study. Aniline-based BA forms a condense layer while antibody could automatically immobilize on the surface of the electrode. Compare to traditional self-assembled monolayer method, the electrodeposition process dramatically reduces the modification time from days to seconds. It also enhances the immobilized efficiency from 95 to 408 (ng/cm^2^) with a strong preference being exhibited for shorter aniline-based linkers.

## 1. Introduction

Biosensors have attracted a great deal of attention because of their potential to be developed into tools capable of rapidly diagnosing various diseases [1]. Remarkable sensors include surface plasmon resonance (SPR) [2], silicon nanowire field-effect transistor (SiNW-FET) [3,4,5], mid-infrared (mid-IR) [6] spectroscopy and quartz crystal microbalance (QCM) [7] devices. Compared with other systems, the QCM chip has an exceptionally robust structure and ionic-strength-independence characteristics and enables the measurement of changes in mass at the molecular level, distinguishing the QCM system as one of the most attractive biosensors [8].

The suitability of quartz crystal microbalance (QCM)-based biosensors for immunosensors is favorable in fields such as drug discovery [9], molecular interaction [10], and clinical diagnosis [11] because of their simplicity and rapid, label-free detection. This biosensing system can easily measure the variation of mass change to the nanogram scale with high precision [12]. QCM responds to the binding of analytes to the sensing layer [13] by changing frequency, which usually immobilizes the bioreceptors capable of capturing certain targets through site-specific recognition. Antibodies (Abs) are one of the most powerful receptors for biosensing, and their immobilization strategy is vital to success [14]. A proper immobilization method could contribute to higher Ab density and thus increase the antigen binding capacity by more than 2–3 times [15,16]. Compared with physical adsorption, chemical bonding enhances attachment stability and is more frequently used. Covalent bonds are mostly formed from hydrophilic site-chain residues including amine, thiol, carboxyl, and hydroxyl moieties at the Ab surface [17,18]. Despite the excellent density improvement demonstrated by these methods, the attachment of Abs to the surface of the electrode leads to random blockages of the binding site, which inhibits antigen recognition. Studies have revealed that the orientation-controlled immobilization of Ab on sensors can enhance the detection signal by 2 to 200-fold [19]. In addition to the application of protein A or protein G, chemically or physically approaching the *N*-glycan portion of the Fc region is another feasible strategy for orientation-controlled Ab immobilization. The orderly arrangement of Ab on the sensing layer optimizes the immunosensing performance [20]. 

Boronic acids (BAs) can bind to 1,2-diols or 1,3-diols, and exhibit a high affinity for forming boronate ester complexes. Such features have been adopted for the separation of carbohydrates, the detection of glycoproteins, molecular recognition, and carbohydrate-related biosensing [21]. BA demonstrates excellent potential for Ab immobilization, with advantages such as performing self-assembly reactions with Ab and the orientation-controlled arrangement of Ab as a result of the exposure of its antigen-binding site. BA derivatives are demonstrably excellent candidates for immunosensor linkers [22]. The method for effectively conjugating BA derivatives on the electrode surface remains crucial in sensor fabrication.

To date, several techniques for BA conjugation have been developed, although their efficacy is frequently challenged. Among them, a two-step strategy without extensive synthetic requirements is commonly employed to introduce carboxylic acid residues on the electrode and couple them with boronate-containing amino groups (amino-BAs) [23,24,25]. Because a multistep reaction often correlates to long reaction time and low production yield, an effective process for introducing functional groups on the electrode surface is desired. A well-known method that offers a one-step modification process is the adoption of a self-assembled monolayer (SAM) from the spontaneous reaction between the sulfhydryl group and Au surface. SAM molecules were first randomly attached to an Au surface during the physisorption step, after a much slower chemisorption reaction formed an oriented monolayer through Au-S-bond formation [26]. Although an SAM layer can be obtained through easy preparations, the reaction time often takes hours to days [27]. Therefore, developing a simple and effective BA modification process for fabricating a sensing device with a high density and stably bound Abs is warranted.

## 2. Materials and Methods

### 2.1. Materials and Reagents

Deionized (DI) water (>18 MΩ·cm) obtained from a purification system (Millipore Synergy, Millipore Co., Billerica, MA, USA) was used throughout the experiments. The phosphate buffered saline (PBS) consists of NaCl (137 mM), KCl (2.7 mM), Na_2_HPO_4_ (10 mM) and KH_2_PO_4_ (2 mM) in NaOH at pH 7.4. The chemicals 4-aminobenzoic acid, 2-amino-2-hydroxymethyl propane-1,3-diol(Tris), 2-(*N*-morpholino) ethanesulfonic acid (MES), tetrahydrofuran (THF), hydroxyl-benzotriazole, *N*-(3-dimethylaminopropyl)-*N*′-ethylcarbodiimide (EDC), *N*,*N*-dimethyl-1,4-phenylene diamine (DMPD), triethylamine (TEA), tris(2-chloroethyl) phosphate (TCEP), 3-mercapto-phenylboronic acid (BA-C_0_-SH) (Sigma-Aldrich, St. Louis, MO, USA), dichloromethane (DCM), 4-dimethylaminopyridine (DMAP), ethyl acetate (EA) (J. T. Baker, Radnor, PA, USA), QCM chips (P-chip AU25, ANT Technology Co., Taipei, Taiwan) were obtained from the indicated suppliers. BA-C_2_-AN, BA-C_7_-AN, BA-C_14_-AN, BA-C_5_-SH and BA-C_7_-SH were synthesized in this work (the detailed synthesis protocols are described in the Supplementary Information).

### 2.2. Instruments

The ^1^H-NMR (300 MHz) and ^13^C-NMR (75.4 Hz) spectra for solution *d*_6_-DMSO were recorded on a DRX-300 NMR (Bruker, Billerica, MA, USA). ESI (electrospray ionization) high resolution mass spectrometry (ESI-HRMS) was carried out on an Ultra-High Resolution Qq-Time-of-Flight IMPACT HD instrument (Bruker).

### 2.3. Self-Assembly (SAM) Gold Electrode Modification

Self-assembled (SAM) thiol modification on a gold surface was performed as follows: disulfide bond compounds are dissolved in 50 μL DMSO (5mM) and aqueous tris(2-carboxyethyl)phosphine (TCEP, 1 eq) is added as reducing agent in order to break the disulfide bonds. The mixture is dropped on the surface of gold for 24 h at room temperature in the dark. After the modification, the gold surface is washed with DMSO and water.

### 2.4. Electrochemical Cleaning Process

The gold surface of an electrode is cleaned by a potential-sweep method in order to wash out the impurities on the surface. The electrode is soaked in 500 mM KOH solution and connected to a potentiostat/galvanostat (660D, CH Instruments Inc., Austin, TX, USA); stationary platinum is used as a counter electrode and Ag/AgCl as a reference electrode. The electrode potential is swept from 0.2 V to −2 V twice at scan rate 0.1 V/s. After the mentioned cleaning process, the surface is washed with water.

### 2.5. Electrodeposition

Aminophenyl functional compounds (10 mM in final solution) are dissolved in 2 mL DMSO stirred for 5 min then added 8 mL H_2_O and stirred on ice for another 5 min. After adding 1 mL 1 M HCl_(aq)_, the solution is mixed with 1 mL sodium nitrate_(aq)_ (1 eq) and stirred for 20 min on ice. The gold electrode is soaked in the aforementioned solution, connected to the potentiostat, and the electrode is scanned from 0 to −1 V (vs Ag/AgCl) at a scan rate 0.25 V/s for 10 cycles. 

### 2.6. Cyclic Voltammetry (CV) and Electrochemical Impedance

CV is recorded by the potentiostat (CHI660) with a three electode configuration. Stationary platinum is used as a counter electrode and Ag/AgCl as a reference electrode. The current response is detected in aqueous solution containing 10 mM K_4_[Fe(CN)_6_] and K_3_[Fe(CN)_6_] dissolved in 1× phosphate buffered saline (PBS) containing NaCl (137 mM), KCl (2.7 mM), Na_2_HPO_4_ (10 mM) and KH_2_PO_4_ (2 mM), pH 7.2). The working electrode is scanned by a scan rate of 0.1 V/s from −0.1 to 0.6 V for CV. Electrochemical impedance is detected in a frequency range of 10^5^ to 10^−1^ with vibration 5 × 10^−3^ at 0.2 V by a scan rate of 0.1 V/s.

### 2.7. QCM Measurement 

Modified QCM chips (P-chip Au25, f_0_ = 9 MHz) are operated at a fundamental frequency (9 MHz) with an ADS analyzer system (ANT Technology Co.). The entire system including flow cell, injection valve and peristaltic pump is connected with Teflon tubing (1/16 inch). In the beginning, the tube is rinsed with 1 M NaOH for 20 min at a 50 mL/min flow rate. After then, 1 M HCl flows through the tube in the same flow rate for 20 min. The entire system is rinsed by double-distilled water for an hour and changed to PBS buffer for another 30 min. The modified QCM chip is placed on the sensor and flow cell is filled with PBS buffer in a flow rate of 25 mL/minute until the frequency is balanced (the change of the frequency is less than 5 Hz in 500 s). Although the volume of the injection valve is 100 μL, over 200 μL of injected sample volume is still required to avoid the generation of bubbles in the flow cell.

## 3. Results and Discussion

**Figure 1:**
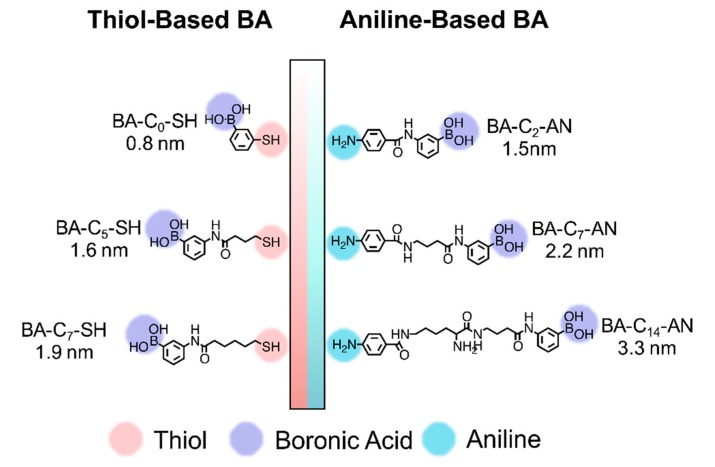
Derivatives of the BA spacer: aniline-based BA modified through electrodeposition and the thiol-based BA modified through SAM on a QCM chip.

In this study, aniline-based BA derivatives were synthesized and used for electrode modification through electrodeposition. QCM was employed to evaluate the immobilization efficiency of these compounds and compare Ab loading capacities. 3-Aminophenylboronic acid is first conjugated with chains of varying lengths, such as aminobutyric acid and lysine, and aniline is then added to the BA derivatives (referred to as BA-C_2_-AN, BA-C_7_-AN, and BA-C_14_-AN in Figure 1, the organic synthesis process in Schemes S1–S3, NMR and HR-MS in Figures S2–S4). Aniline-based BAs were introduced into acidic solution, resulting in the formation of aryldiazonium species, which were subsequently reduced to radical species through an electrochemical process. An aryl radical grafted to the Au surface, with a quick reaction forming a BA layer on the QCM chip [28]. The duration of the whole electrodeposition process was shorter than 10 min. 

To compare the modification efficiency, reaction time, and length-dependent capacity of Abs, we also synthesized thiol-based BAs (BA-C_0_-SH, BA-C_5_-SH, BA-C_7_-SH), as illustrated in Figure 1 (the organic synthesis process showed in Schemes S4 and S5, NMR and HR-MS in Figures S5 and S6), that were used in the disulfide bond formation. After cleaving the disulfide bond through tris(2-carboxyethyl)phosphine (TCEP), the thiol-based BA derivatives were deposited on the Au electrode surface and retained there for 24 h. The modification process was verified by using CV and electrical impedance measurements.

**Figure 2:**
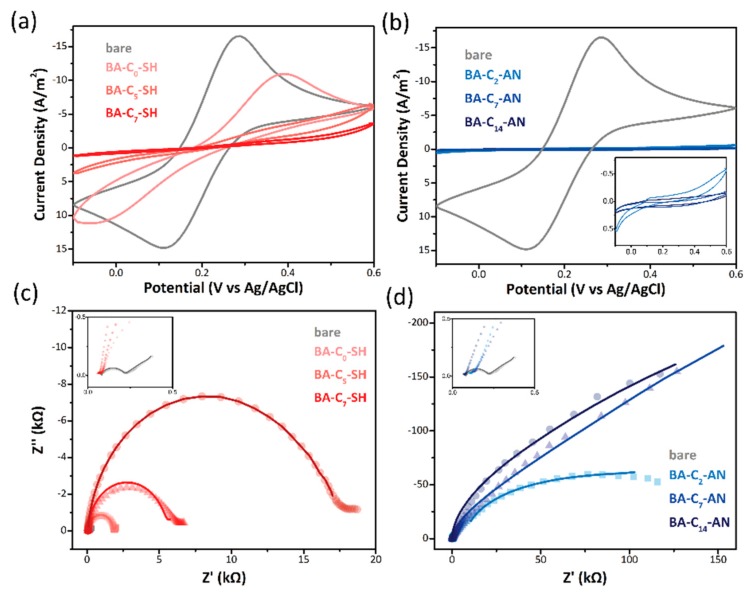
CV of an Au electrode of (**a**,**b**) bare (grey), thiol-based (red) BAs and aniline-based BA derivatives were in 10 mM ferricyanide. Electrical Impedance of thiol-based (**c**) (red) and aniline-based (**d**) (blue) BA derivatives were fitted by the Randles equivalent where experiment result showed in dot and the fitted curve in line.

The CV of the bare Au electrode, modified thiol-based BA (Figure 2a), and aniline-based BA (Figure 2b) are displayed in results detailing the modification efficiency. The potential was swept between −0.1 V and 0.6 V at a scan rate of 100 mV/s in the presence of a 10 mM solution of [Fe (CN)6]-4/−3. The grey line signifies the points at which the bare Au electrode revealed high oxidation and reduction current peaks. Although the surface was coated with BA derivatives, the redox current peaks were reduced presumably because of the decreased exposure area of the electrode. As displayed in Figure 2b, an aniline-based BA coating significantly reduced the redox current, demonstrating high modification efficiency and coverage density on the QCM Au electrode. Conversely, the thiol-based BA only slightly decreased the redox peak. The length of the BA derivative also influenced the redox peak intensity. By increasing the compound length, the hydrophobic carbon chain was more efficient at blocking ion exchange through the electrode surface, resulting in the low current intensity in the redox signal. This behavior was commonly observed in electrodes derived through both modification methods. The Nyquist plots of the BA derivatives are shown in Figure 2c,d. The bare gold electrode reveals faster electron transfer by exhibiting smaller semicircular diameter. In both thiol-based and aniline-based BA impedance plot, semicircular diameter enlarged with the increasing length of the spacer, suggesting the higher electron transfer resistance that might due to the increasing thickness and density of the modified surface [29,30]. Impedance results is fitted with Randles equivalent where electron-transfer resistance (R_ct_), electrolyte resistance (R_s_) and constant phase element (CPE) are presented in Table 1.

In order to ensure the immobilization ability of BA residual, a strong blocking compound, sorbitol, is adding to the system under QCM surveillance (Figure S1). Sorbitol forms strong diol complex with BA blocking the immobilization active site of the spacer. After blocking BA active site, there is less than 5 Hz frequency change during the antibody injection in QCM result implying only small amont of antibody binds to the electrode surface. 

**Figure 3:**
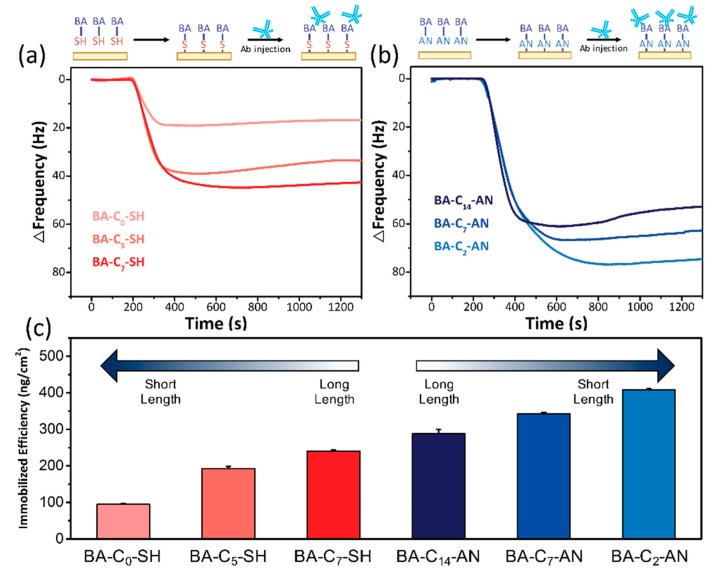
Recorded frequency responses to 25 µg/mL Ab of (**a**) a thiol-based BA spacer (red lines) and (**b**) an aniline-based BA spacer (blue lines) modified by a QCM chip in a 1× PBS buffer under real-time surveillance. (**c**) Comparison of the length-dependent effects on Ab-immobilization efficiency between the electrodeposition (blue) and SAM (red) methods.

Figure 3a,b illustrate the immobilization efficiency of the two modification methods through QCM monitoring. After filling with PBS buffer, the flow cell was injected with 100 μL of 25 μg/mL mouse IgG into the flow. As the BA length increased, the change of frequency also increased in SAM (Figure 3a). Under the same loading condition, BA-C_0_-SH caused a small frequency change (17.3 ± 0.4 Hz), and increasing chain lengths in BA-C_5_-SH (35.1 ± 1.1 Hz) and BA-C_7_-SH (43.9 ± 0.5 Hz) also improved Ab capacity. This phenomenon has been observed in other studies, in which longer linker lengths demonstrated a greater capacity for the immobilization of target proteins.

Surprisingly, however, aniline-based BA exhibited the opposite behavior (Figure 3b). BA-C_2_-AN, with the shortest BA length, was able to induce a prominent frequency change (74.5 ± 0.5 Hz) through the electrodeposition method, whereas the medium-length BA-C_7_-AN produced only a minor response signal (62.5 ± 0.8 Hz) and the longest BA-C_14_-AN only produced 52.6 ± 2.1 Hz. The greater binding capacity of Ab through electrodeposition methods than through SAM is consistent with the findings of our previous study [7]. This unexpected dependency of Ab binding capacity on linker length has never been reported before.

From the variations in frequency, we could infer the mass accumulation on the Au electrode by using the Sauerbrey equation, where ΔF represents the measured change in frequency, F_R_ is the resonant frequency of the quartz crystal (9 × 10^6^ Hz in this case), ΔM is the deposited mass (g), A signifies the electrode area on the quartz crystal (cm^2^), ρ_Q_ is the density of quartz (2.65 g/cm^3^), μ_Q_ indicates the shear modulus of quartz (2.95 × 10^11^ g/cm s), and C_Q_ is the constant for quartz (−2.26 × 10^−6^):

$$\Delta F=-2{F_{R}}^{2}\frac{\Delta M}{\sqrt{\rho_{Q}\mu_{Q}}}A=C_{Q}{F_{R}}^{2}\frac{\Delta M}{A}$$

After the total mass of Ab is deduced, we can also determine the efficiency of immobilized Abs. Assuming a plane surface, the surface area of the Au electrode was 1.35 × 10^−1^ cm^2^. The frequency change, mass accumulation, and immobilization efficiency are summarized in Table 2 and Figure 3c. The highest Ab-immobilization efficiency was 408.1 ng/cm^2^, produced with the contribution of BA-C_2_-AN. This aniline-based linker with a shorter chain demonstrated greater efficiency for Ab immobilization, whereas the traditional SAM process for modification of the electrode surface exhibited the opposite trend. A longer spacer provided stronger chain–chain interactions that increased the high density of orderly alignment [31]. Also, in monolayer cases, attachment to a long, flexible, and adjustable chain immobilized Ab in an upright position. This type of situation can improve the capacity of Ab on limited electrode space. Chen et al. described the same result in the BA spacers of an Fc–fused-lectin-protein microarray [32]. Other immunosensor-related articles have also investigated length-dependent efficiency and reached the same conclusion that extended length markedly improves Ab-immobilization efficiency [33]. 

## 4. Discussion

**Figure 4:**
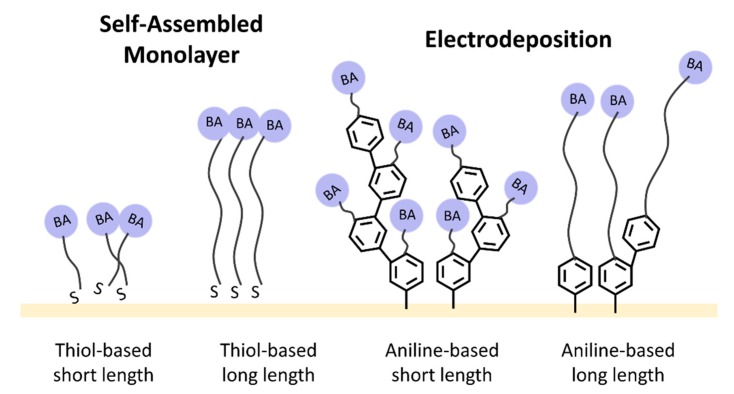
Derivatives of BA spacers: aniline-based BA modified through electrodeposition and thiol-based BA modified through SAM on a QCM chip.

A logical explanation for this result is that aniline-based BA is not a uniform monolayer deposited on the Au surface. A relevant phenomenon was described by Belanger et al. in a demonstration of the formation process of electrografted polyfilm [34,35]. Aryl species deposited on BA monomers formed radical species under diazonium ion formation conditions and attached to the electrode surface through a redox current. Remaining aryl groups were grafted to the phenyl group of the initial layer and more radically attacked the grafted chain. The reaction extended the length of the polymer and also increased the occurrence of BA residue formation. The deposited layer exhibited continual growth until a certain level was reached at which the redox current was unable to provide sufficient energy for bond formation. As a result of the electrografting process, the short spacer layer formed a higher BA-residue density, thus increasing the number of available binding sites for carbohydrates. It seems that this multiple-BA grafting structure may also lead to greater steric effects in circumstances where Abs require a longer reaction time. The association time of Abs in the QCM of BA-C_2_-AN was slightly longer than it was for others (Figure 3b), but this was quickly overcome; moreover, QCM demonstrated the highest immobilization efficiency overall. In polymerization, short spacers provided sufficient length for Ab to be situated upright on the electrode. Such positioning enabled more orderly alignment and condensed packing, leading to high immobilization efficiency. A comparison of the length-dependent efficiency in different modification methods is illustrated in Figure 4.

## 5. Conclusions

In conclusion, we have developed a rapid-construction BA layer with high capacity on the Au electrode of a QCM chip through electrodeposition. In comparison with the SAM, a simple one-step method of depositing aniline-based BA on the Au surface reduced a modification time from 24 hours to only 10 seconds. Also, a four times higher Ab-immobilization efficiency (408.1 ng/cm^2^) was achieved with a short link aniline BA. Our findings also demonstrate that shorter spacers lead to higher Ab immobilization efficiency. This study provides a new protocol for the effective fabrication of biosensors for various applications.

## Figures and Tables

**Table 1:** Fitted circuit elements of BA derivatives on QCM electrodes.

Modification Method	Bare Electrode	Self-Assembled Monolayer Modified			Electrodeposition Modified		
Compound Name		BA-C_0_-SH	BA-C_5_-SH	BA-C_7_-SH	BA-C_2_-AN	BA-C_7_-AN	BA-C_14_-AN
R_s_ (kΩ)	0.085	0.081	0.087	0.087	0.126	0.144	0.115
R_ct_ (kΩ)	0.123	1.735	4.939	12.651	27.927	43.577	63.454
CPE (µF)	2.8	0.641	0.784	0.590	1.359	1.19	1.83

**Table 2:** Antibody capacity of a BA-modified QCM chip for the SAM and electrodeposition methods.

Modification Method	Self-Assembled Monolayer Modified			Electrodeposition Modified		
Compound Name	BA-C_0_-SH	BA-C_5_-SH	BA-C_7_-SH	BA-C_14_-AN	BA-C_7_-AN	BA-C_2_-AN
Δ Frequency (Hz)	17.3 ± 0.4	35.1 ± 1.1	43.9 ± 0.5	52.6 ± 2.1	62.5 ± 0.8	74.5 ± 0.5
Total Antibody (ng)	12.8	26	32.5	38.9	46.2	55.1
Immobilization efficiency (ng/cm^2^)	94.8	192.6	240.7	288.1	342.2	408.1

## Supplementary Materials

The following are available online at http://www.mdpi.com/1424-8220/19/1/28/s1. Figure S1: Real-time response of a QCM sensor by injecting sorbitol and mouse IgG. Figure S2: ^1^H NMR, ^13^C NMR, and ESI+ HRMS spectra of BA-C_2_-AN. Figure S3: ^1^H NMR, ^13^C NMR, and ESI+ HRMS spectra of BA-C_7_-AN. Figure S4: ^1^H NMR, ^13^C NMR, and ESI+ HRMS spectra of BA-C_14_-AN. Figure S5: ^1^H NMR, ^13^C NMR, and ESI+ HRMS spectra of BA-C_5_-SH. Figure S6: ^1^H NMR, ^13^C NMR, and ESI+ HRMS spectra of BA-C_7_-SH. Scheme S1: Scheme of synthesis of BAC_2_-AN. Scheme S2: Scheme of synthesis of BA-C_7_AN. Scheme S3: Scheme of synthesis of BA-C_14_-AN. Scheme S4: Scheme of synthesis of BA-C_5_-SH. Scheme S5: Scheme of synthesis of BA-C_7_-SH.
