# Supplementary material for: Effective Construction of a High-Capacity Boronic Acid Layer on a Quartz Crystal Microbalance Chip for High-Density Antibody Immobilization
Source: Sensors (Basel). 2018 Dec 21;19(1):28. doi: 10.3390/s19010028 (PMC6339082; doi:10.3390/s19010028)
Supplement: Supplementary file 1 [file sensors-19-00028-s001.pdf]

Supporting Information for:

Communication

Effective Construction of a High-Capacity Boronic Acid Layer on a Quartz Crystal Microbalance Chip for High-Density Antibody Immobilization

Pei-Heng Lin, [1, 2] **Sheng-Cih Huang,** [3] **Kuang-Po Chen,** [3] **Bor-Ran Li** [1,4,*] **and Yaw-Kuen Li** [3, 4,*]

[1] Institute of Biomedical Engineering, College of Electrical and Computer Engineering, National Chiao Tung University, Hsinchu, Taiwan.
[2] Department of Electrical and Computer Engineering, College of Electrical and Computer Engineering, National Chiao Tung University, Hsinchu, Taiwan.
[3] Department of Applied Chemistry, College of Science, National Chiao Tung University, Hsinchu, Taiwan.
[4] Center for Emergent Functional Matter Science, National Chiao Tung University, Hsinchu, Taiwan
* Correspondence: liborran@g2.nctu.edu.tw, ykl@g2.nctu.edu.tw

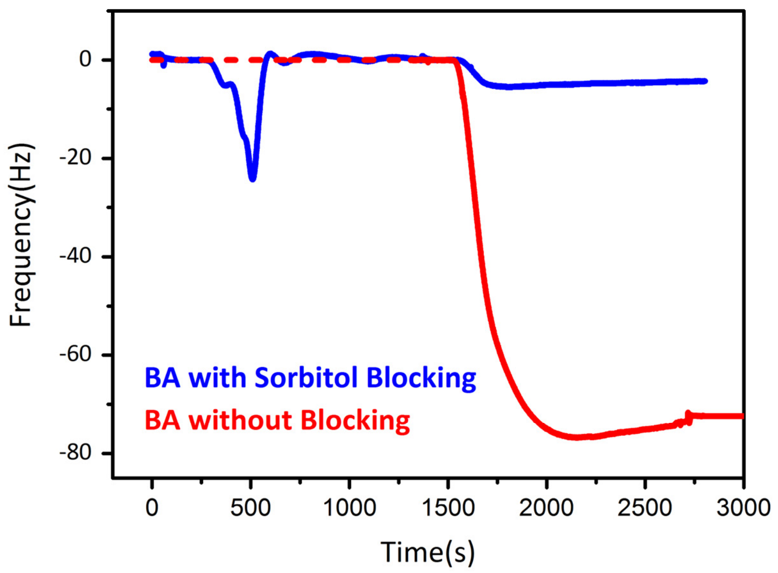

Figure S1. Real-time frequency shifts of a QCM sensor by injecting sorbitol and mouse IgG (25 μg/mL) into modified BA-C_2-AN without injecting sorbitol (red line) and BA-C_2-AN with sorbitol blocking (blue line).

Synthesis of boronic derivatives

3-(4-aminobenzamido)phenylboronic acid (BA-C2-AN)

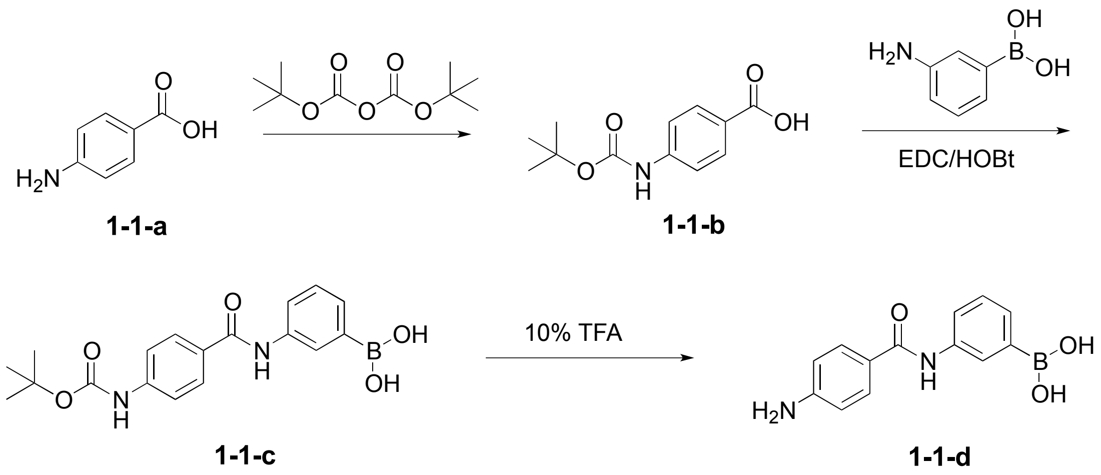

Scheme S1. Scheme of synthesis of 3-(4-aminobenzamido)phenylboronic acid (BA-C2-AN)

4-aminobenzoic acid **(1-1-a)** is added into 1-4-dioxane (40 mL) and aqueous NaOH (1M,40 mL), the mixture is cooled down to 0 °C and stirred for 15 minutes. Di-tert-butyl dicarbonate (1.2eq) dissoveled in 1-4-dioxane (20 mL) is added dropwise within 10 minutes and the reaction is warm up to room temperature stirred for another 12 hours. The resulting mixture is quenched by 1 M HCl_{aq} to pH=2.5 and extracted with ethyl acetate(EA) (250 mL), 1 M HCl_{aq} and brine. The combined organic layer is dried over anhydrous $MgSO_4$ and filtered, and solvents are evaporated under reduced pressure. Column chromatography (EA/n-hexane, 1:1, v/v) gives the pure product **(1-1-b)**. Yield 90%

(1-1-b) is added into anhydrous dimethylforamide (DMF) (10mL) stirred for 10 minutes. 1-Ethyl-3-(3-dimethylaminopropyl)carbodiimide (EDC, 1.1 eq) and Hydroxybenzotriazole (HOBt, 1.5 eq) are added into reactant subsequently under dry N_2 atmosphere stirred for 2 hours. Dissolved in DMF (10 mL), 3-aminophenylboronic acid is mixed in the reaction with triethylamine (1 eq) as base. After stirred for 12 hours, the reaction can be confirmed by TLC in ninhydrin stain. The resulting mixture is added 1mL into 50mL centrifuge tube and fulfilled by 0.1 M HCl_{aq} and centrifugared for 3 times each 20 minutes. The solid suspension is dissolved in dichloromethane(DCM) and Methanol solvent mixture. Column chromatography (DCM /Methanol, 10:1, v/v) gives the pure

product **(1-1-c)**. Yield 80%.

Compound **(1-1-c)** is dissolved in DCM (20 mL) and trifluoroacetic acid (2 mL) is added dropwise into the mixture under N_2 atmosphere stirred for 8 hours. Solvents and acid are evaporated under reduced pressure and the pure product 3-(4aminobenzamido)phenyl boronic acid (BA-C_2-AN **(1-1-d)**) can be collected. Yield 90%.

^{1}H NMR (300MHz, d_6-DMSO) δ 8.02 (s, 1H), 7.82-7.79 (m, 3H), 7.50 (d, J = 7.43 Hz, 1H), 7.28 (t, J = 7.43, 1H), 6.77 (d, J = 8.4Hz, 2H). ^{13}C NMR (75.4 MHz, d_6-DMSO) δ 164.6, 147.4, 138.2, 128.9, 128.7, 127.0, 126.1, 124.1, 122.0, 114.7. HRMS(ESI) exact mass calcd. for $C_{13}H_{13}BN_2O_3$: m/z 257.1094 ([M+H]+); found: m/z 257.1099([M+H]+).

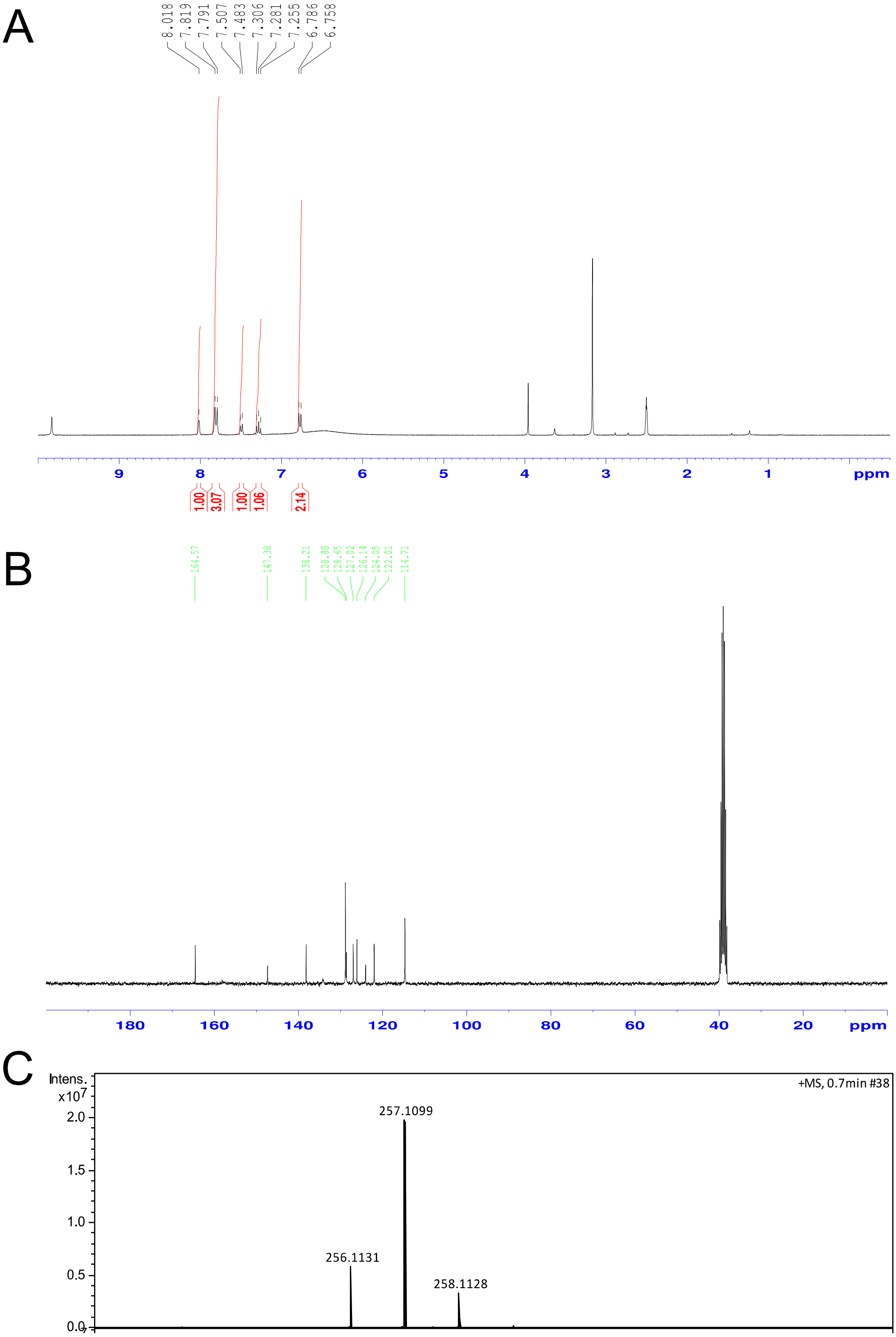

Figure S2. (a) ^{1}H NMR (b) ^{13}C NMR, and (c) ESI+ HRMS spectra of BA-C$_2$-AN

3-(4-(4-aminobenzamido)butanamido)phenylboronic acid (BA-C$_7$AN)

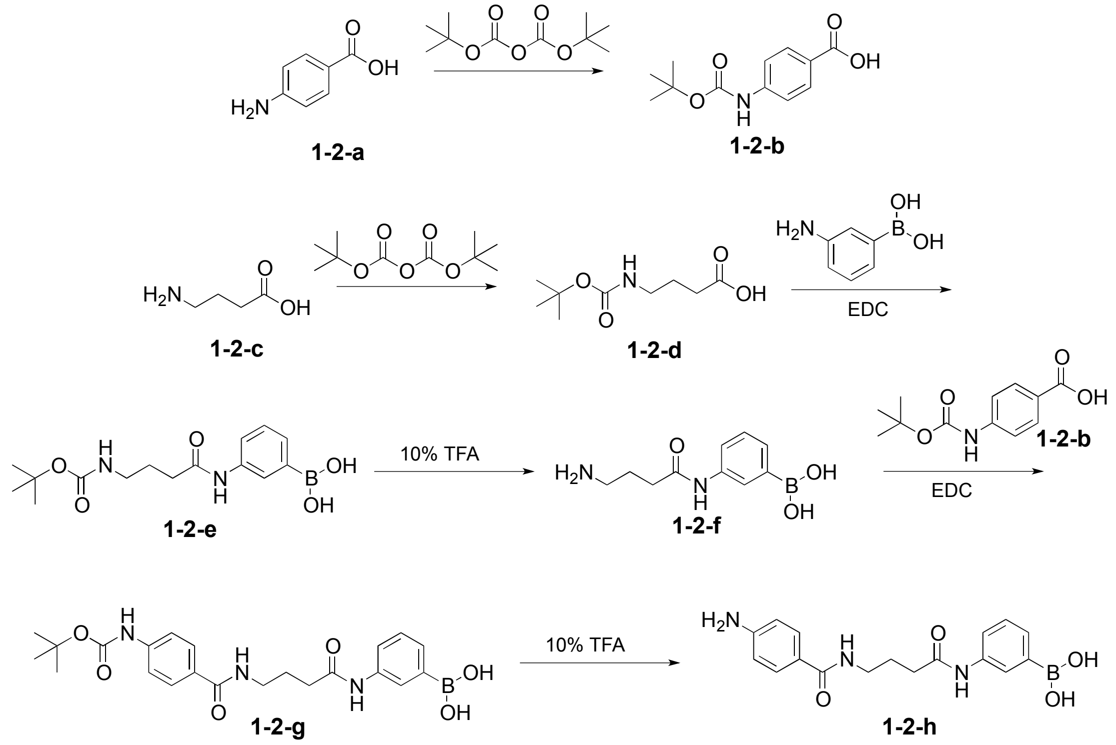

Scheme S2. Scheme of synthesis of 3-(4-(4-aminobenzamido)butanamido phenylboronic acid (BA-C$_7$AN)

4-aminobenzoic acid **(1-2-a)** is added into 1-4-dioxane (40 mL) and aqueous NaOH (1M,40 mL), the mixture is cooled down to 0°Cand stirred for 15 minutes. Di-tert-butyl dicarbonate (1.2eq) dissoveled in 1-4-dioxane (20 mL) is added dropwise within 10 minutes and the reaction is warm up to room temperature stirred for another 12 hours. The resulting mixture is quenched by 1 M HCl_{aq} to pH=2.5 and extracted with EA (250 mL), 1 M HCl_{aq} and brine. The combined organic layer is dried over anhydrous $MgSO_4$ and filtered, and solvents are evaporated under reduced pressure. Column chromatography (EA/n-hexane, 1:1, v/v) gives the pure product **(1-2-b)**. Yield 90%.

γ-Aminobutyric acid **(1-2-c)** is added into 1-4-dioxane(40 mL) and aqueous NaOH (1 M,40 mL), the mixture is cooled down to 0°Cand stirred for 15 minutes. Di-tert-butyl dicarbonate (1.2eq) dissoveled in 1-4-dioxane (20 mL) is added dropwise within 10 minutes and the reaction is warm up to room temperature stirred for another 12 hours. The resulting mixture is quenched by 1M HCl_{aq} to pH = 2.5 and extracted with EA (250 mL) ,1M HCl_{aq} and brine. The

combined organic layer is dried over anhydrous $MgSO_4$ and filtered, and solvents are evaporated under reduced pressure. Column chromatography (EA/n-hexane, 1:1, v/v) gives the pure product **(1-2-d)**. Yield 90%.

Product **(1-2-d)** is added into anhydrous DMF (10mL) stirred for 10 minutes. Commercial 3-aminophenylboronic acid (1 eq) is sebsequently added into the mixture and stirred for another 10 minutes.Coupling reagent , EDC (1.5eq), is added into reactant under dry N_2 atmosphere stirred for 12 hours at 4°C. The reaction can be confirmed by TLC in ninhydrin stain. The resulting mixture is added a small amount of toluene in order to azeotrope with DMF and evaporated under reduced pressure. Column chromatography(DCM /Methanol, 10:1, v/v) gives the pure product **(1-2-e)**. Yield 85%.

Compound **(1-2-e)** is dissolved in DCM (20 mL) and trifluoroacetic acid (2 mL) is added dropwise into the mixture under N_2 atmosphere stirred for 8 hours. The reaction is confirmed by TLC in ninhydrin stain. Solvents and acid are evaporated under reduced pressure and the pure product **(1-2-f)** can be collected. Yield 90%.

Product **(1-2-f)** is added into anhydrous DMF (10 mL) stirred for 10 minutes. EDC (1.1eq) and Hydroxybenzotriazole (HOBt, 1.5eq) are added into reactant subsequently under dry N_2 atmosphere stirred for 2 hours. Dissolved in DMF (10mL), advenced product **(1-2-b)** is mixed in the reaction with triethylamine (1 eq) as base. After stirred for 12 hours, the reaction can be confirmed by TLC in ninhydrin stain. The resulting mixture is added 1mL into 50 mL centrifuge tube and fulfilled by 0.1M HCl_{aq} and centrifugared for 3 times each 20 minutes. The solid suspension is dissolved in DCM and Methanol solvent mixture. Column chromatography (DCM /Methanol, 10:1, v/v) gives the pure product **(1-2-g)**. Yield 70%.

Compound **(1-2-g)** is dissolved in DCM (20 mL) and trifluoroacetic acid (2 mL) is added dropwise into the mixture under N_2 atmosphere stirred for 8 hours. Solvents and acid are evaporated under reduced pressure and the pure product 3-(4-(4-aminobenzamido)butanamido)phenylboronic acid (BA-C_7-AN, **(1-2-h)**) can be collected. Yield 90%.

^{1}H NMR (300MHz, d_6-DMSO) δ 7.81 (s, 1H), 7.70 (d, J = 7.95 Hz, 1H), 7.63 (d, J = 8.4 Hz, 2H), 7.44 (d, J = 7.95 Hz, 1 Hz), 7.23 (t, J = 7.95 Hz, 1H), 6.68 (d, J =

8.4 Hz, 2H), 3.28 - 3.22 (m, 2H), 2.35 - 2.30 (t, J = 7.28 Hz, 2H), 1.82 - 1.77 (t, J = 7.28 Hz, 2H). ^{13}C NMR (75.4 MHz, d_6-DMSO) δ 171.4, 166.5, 147.0, 138.9, 129.3, 129.2, 128.1, 125.6, 121.6, 116.1, 39.2, 34.4, 25.9. HRMS(ESI) exact mass calcd. for $C_{17}H_{20}BN_3O_4$: m/z 342.1623 ([M+H]+); found: m/z 342.1633([M+H]+).

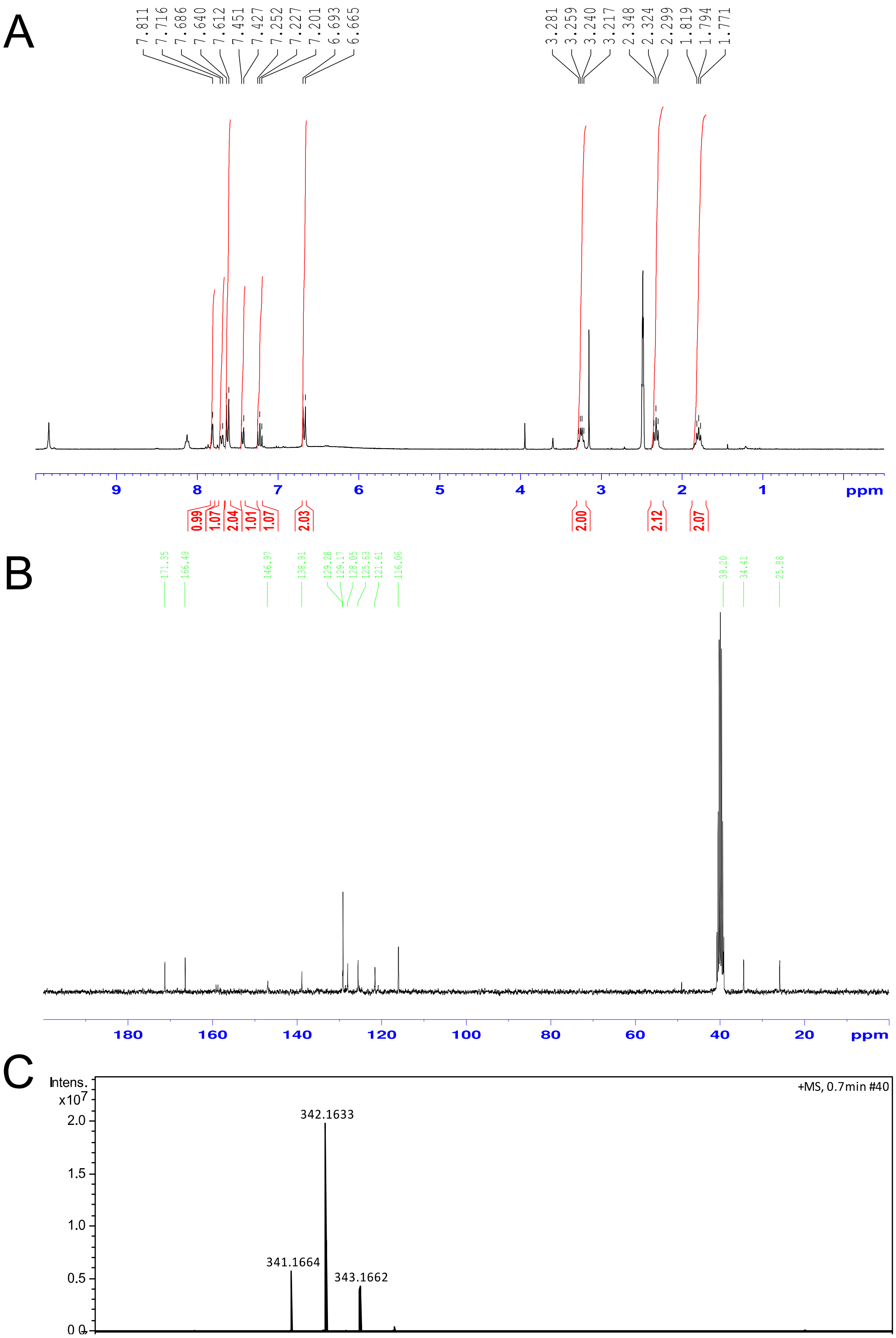

Figure S3. (a) ^{1}H NMR (b) ^{13}C NMR, and (c) ESI+ HRMS spectra of BA-C_7-AN

3-(4-(2-amino-6-(4-aminobenzamido)hexanamido)butanamido)phenylboronic acid (BA-C14-AN)

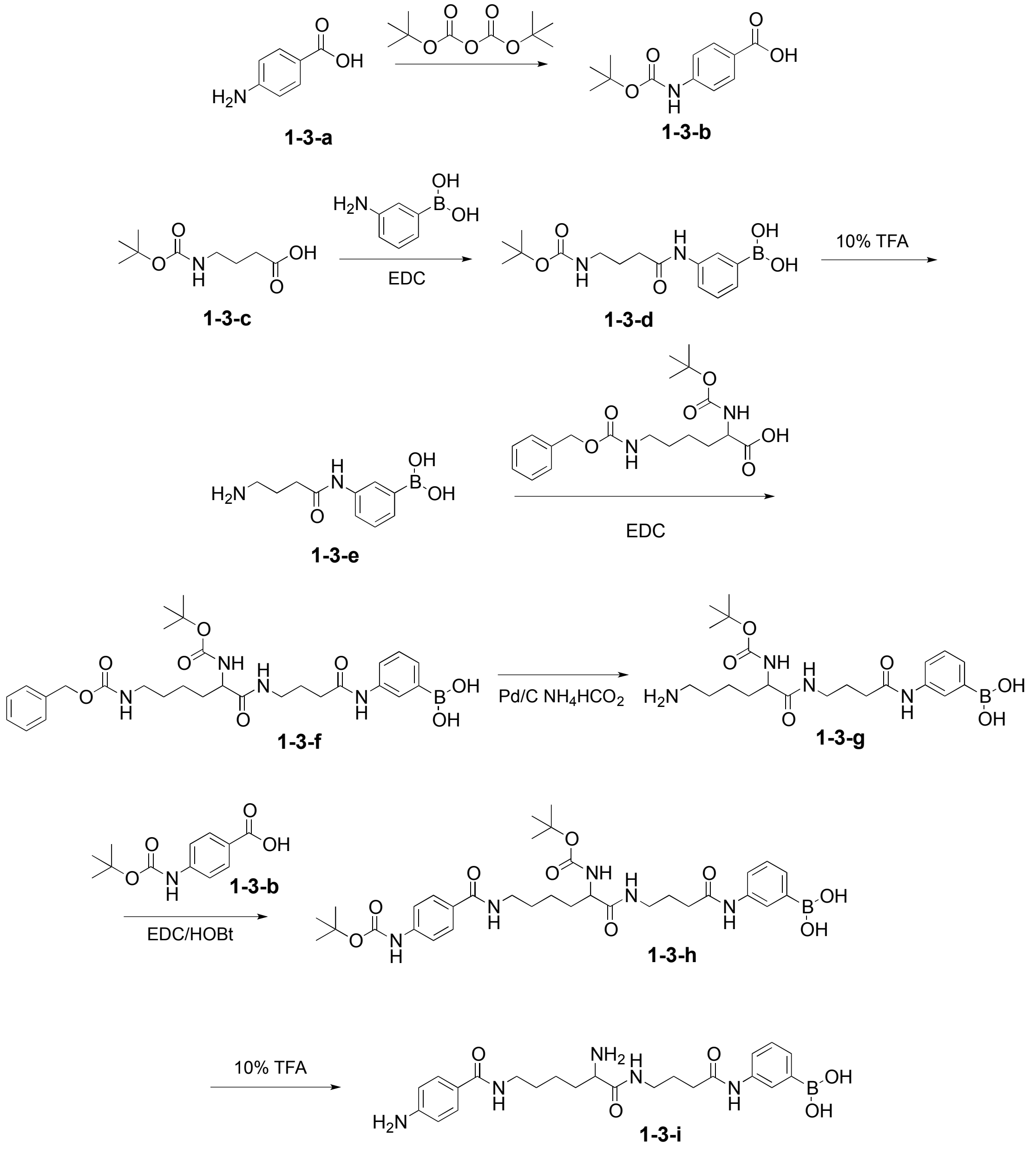

Scheme S3. Scheme of synthesis of 3-(4-(2-amino-6-(4-aminobenzamido) hexanamido)butanamido)phenylboronic acid (BA-C_{14}-AN)

4-aminobenzoic acid **(1-3-a)** is added into 1-4-dioxane (40 mL) and aqueous NaOH (1M,40 mL), the mixture is cooled down to 0°C and stirred for 15 minutes. Di-tert-butyl dicarbonate (1.2eq) dissoveled in 1-4-dioxane (20 mL) is added dropwise within 10 minutes and the reaction is warm up to room temperature stirred for another 12 hours. The resulting mixture is quenched by

1 M HCl_{aq} to pH=2.5 and extracted with EA (250 mL), 1 M HCl_{aq} and brine. The combined organic layer is dried over anhydrous $MgSO_4$ and filtered, and solvents are evaporated under reduced pressure. Column chromatography (EA/n-hexane, 1:1, v/v) gives the pure product **(1-3-b)**. Yield 90%.

Product **(1-3-c)** is added into anhydrous DMF (10 mL) stirred for 10 minutes. Commercial 3-aminophenylboronic acid (1 eq) is sebsequently added into the mixture and stirred for another 10 minutes. Coupling reagent, EDC (1.5eq), is added into reactant under dry N_2 atmosphere stirred for 12 hours at 4°C. The reaction can be confirmed by TLC in ninhydrin stain. The resulting mixture is added a small amount of toluene in order to azeotrope with DMF and evaporated under reduced pressure. Column chromatography(DCM /Methanol, 10:1, v/v) gives the pure product **(1-3-d)**. Yield 80%.

Compound **(1-3-d)** is dissolved in DCM (20 mL) and trifluoroacetic acid (2 mL) is added dropwise into the mixture under N2 atmosphere stirred for 8 hours. Solvents and acid are evaporated under reduced pressure and the pure product **(1-3-e)** can be collected. Yield 90%.

Product **(1-3-e)** is added into anhydrous DMF (10 mL) stirred for 10 minutes. Commercial 6-(benzyloxycarbonylamino)-2-(tert-butoxycarbonylamino)hexanoic acid (Boc-Lys(Z)-OH) (1 eq) is sebsequently added into the mixture and stirred for another 10 minutes.Coupling reagent , EDC (1.5eq), is added into reactant under dry N_2 atmosphere stirred for 12 hours at 4°C. The reaction can be confirmed by TLC in ninhydrin stain. The resulting mixture is added a small amount of toluene in order to azeotrope with DMF and evaporated under reduced pressure. Column chromatography(DCM /Methanol, 10:1, v/v) gives the pure product **(1-3-f)**. Yield 80%.

Compound **(1-3-f)** is dissolved in methanol (20 mL) under dry N_2 atmosphere. 10% wt of Pd/C is gently added into the reaction at room temperature and stirred for a while. Ammonium formate (8 eq) is suspended 2 mL of water and mixed with 18 mL methanol. The mixture is injected into the reaction and raised temperature up to 40 °C for 8 hours. After the verification by TLC and ninhydrin stain, the mixture is cooled down to room temperature and filtered by celite in order to remove Pd/C. Redundant methanol is evaporated under reduced pressure and pure product **(1-3-g)** is obtained. Yield 75%.

Product **(1-3-g)** is added into anhydrous DMF (10 mL) stirred for 10 minutes. EDC (1.1eq) and Hydroxybenzotriazole (HOBt, 1.5eq) are added into reactant subsequently under dry N_2 atmosphere stirred for 2 hours. Dissolved in DMF (10mL), advenced product **(1-3-b)** is mixed in the reaction with triethylamine (1 eq) as base. After stirred for 12 hours, the reaction can be confirmed by TLC in ninhydrin stain. The resulting mixture is added 1mL into 50 mL centrifuge tube and fulfilled by 0.1M HCl_{aq} and centrifugared for 3 times each 20 minutes. The solid suspension is dissolved in DCM and Methanol solvent mixture. Column chromatography (DCM /Methanol, 10:1, v/v) gives the pure product **(1-3-h)**. Yield 50%.

Compound **(1-3-h)** is dissolved in DCM (20 mL) and trifluoroacetic acid (2 mL) is added dropwise into the mixture under N_2 atmosphere stirred for 8 hours. Solvents an acid are evaporated under reduced pressure and the pure product 3-(4-(2-amino-6-(4-amino benzamido)hexanamido)butanamido)phenylboronic acid (BA-C_{14}-AN, **(1-3-i)**) can be collected. Yield 90%.

^{1}H NMR (300MHz, d_6-DMSO) δ 7.76 (s, 1H), 7.64 - 7.56 (m, 3H), 7.39 (m, 1H), 7.23 (t, J = 7.65 Hz, 1H), 6.68 (d, J = 8.4 Hz, 2H), 3.76 (t, J = 6.9Hz, 1H), 3.27-3.25 (m, 2H), 2.35 (t, J = 6.9 Hz, 2H), 1.85-1.80 (m, 4H), 1.60-1.55 (m, 2H), 1.41-1.37(m, 2H), 1.27(m, 2H). ^{13}C NMR (75.4 MHz, d_6-DMSO) δ 171.4, 166.5, 147.0, 138.9, 129.3, 129.2, 128.1, 125.6, 121.6, 116.1, 34.4, 25.9. HRMS(ESI) exact mass calcd. for $C_{23}H_{32}BN_5O_5$: m/z 471.2652 ([M+H]+); found: m/z 471.2659([M+H]+).

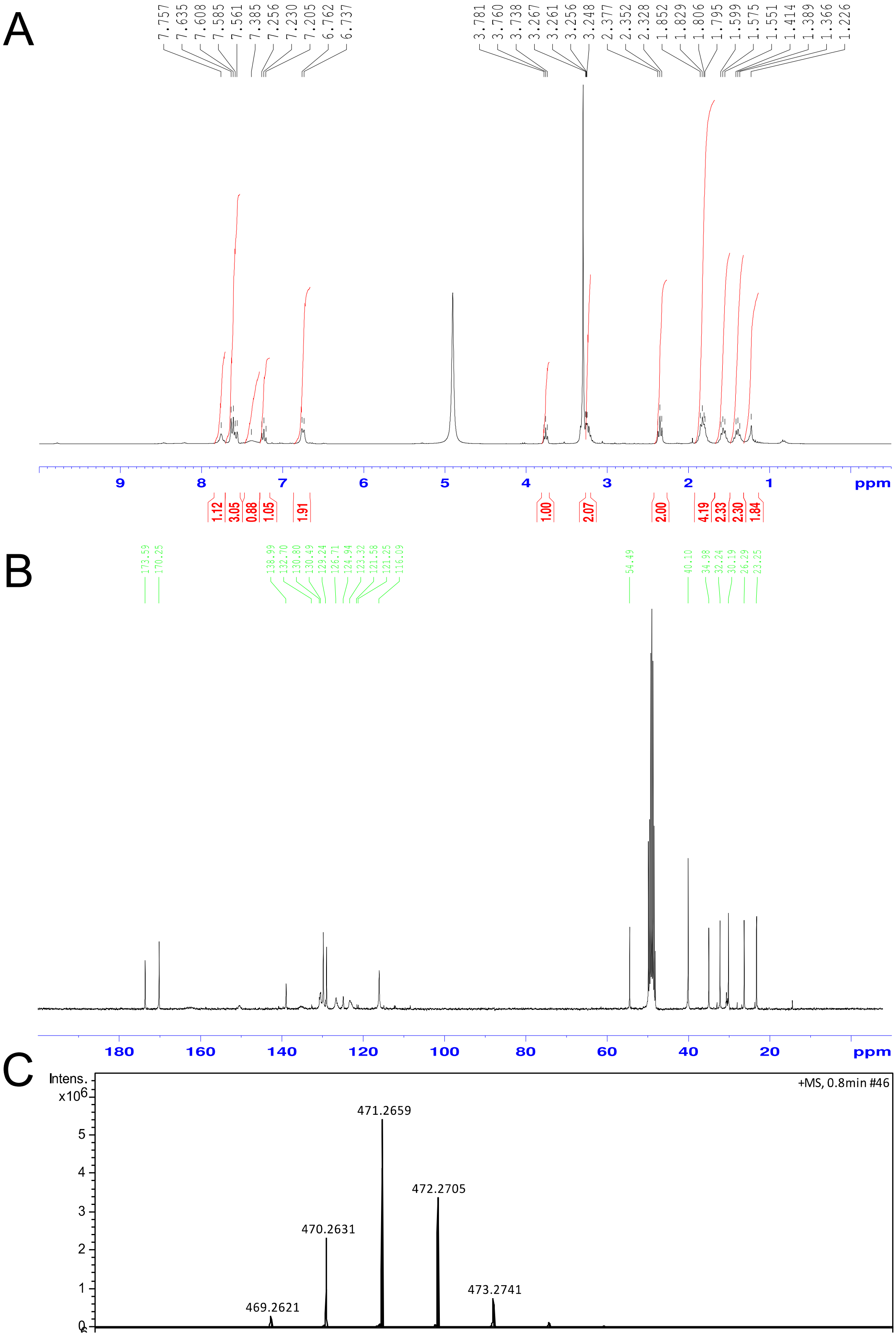

Figure S4. (a) ¹H NMR (b) ¹³C NMR, and (c) ESI+ HRMS spectra of BA-C_{14}-AN

3-(4-mercaptobutanamido)phenylboronic acid (BA-C$_5$-SH)

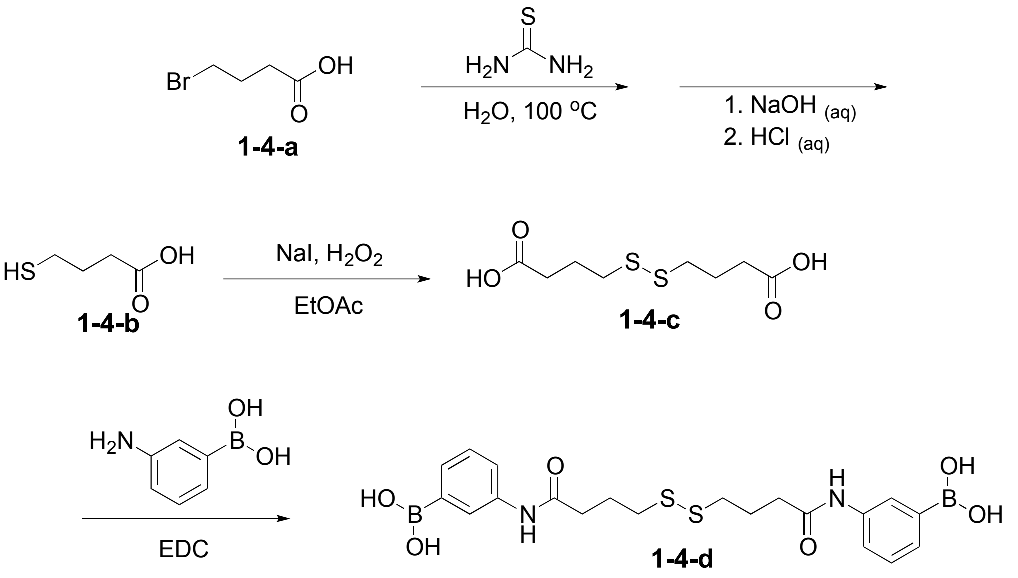

Scheme S4. Scheme of synthesis of 3-(4-mercaptobutanamido)phenylboronic acid (BA-C$_5$-SH)

4-bromobutanoic acid is added into 250mL flask which is contained 30 mL ddH$_2$O with thiourea (1 eq). The reaction is refluxed and heated up to 100 °C under N$_2$ atmosphere for 12 hours. Removing the heat, the mixture is cooled down to room temperature and precipitated white crystal. 2M NaOH$_{(aq)}$ is added dropwise into the mixture, stirred for 2 hours at 0 °C. The resulting mixture is dissolved in ether extracted with 1 M HCl$_{(aq)}$ and brine. The combined organic layer is dried over anhydrous MgSO$_4$ and filtered, and solvents are evaporated under reduced pressure. Column chromatography with ether and hexane gives the pure product **(1-4-b)**.

Compound **(1-4-b)** is added to a solution of 100 mL EA with NaI (0.01eq) stirred vigorously for 5 minutes. The mixture is continues stirred for another 30 minutes after adding 1M H$_2$O$_2$. Confirming the reaction, mixture is extracted with EA, H$_2$O and brine. The combined organic layer is dried over anhydrous MgSO$_4$ and filtered, and solvents are evaporated under reduced pressure. Column chromatography with EA and hexane gives the pure product **(1-4-c)**.

Product **(1-4-c)** is added into anhydrous dimethylforamide (10 mL) stirred for 10 minutes. Commercial 3-aminophenylboronic acid (1 eq) is sebsequently added into the mixture and stirred for another 10 minutes. Coupling reagent,

EDC (1.5eq) is added into reactant under dry N_2 atmosphere stirred for 12 hours at 4°C. The reaction can be confirmed by TLC in ninhydrin stain. The resulting mixture is added a small amount of toluene in order to azeotrope with dimethylforamide and evaporated under reduced pressure. Column chromatography(dichloromethane /Methanol, 10:1, v/v) gives the pure product **(1-4-d)**.

^{1}H NMR (300MHz, d_6-DMSO) δ 7.81 (s, 2H), 7.69 (d, J = 7.8 Hz, 2H), 7.44 (d, J = 7.2 Hz, 2H), 7.28 - 7.20 (m, 2H), 2.78 - 2.73 (m, 4H), 2.43-2.38 (m, 4H), 1.96 - 1.91 (m, 4H). ^{13}C NMR (75.4 MHz, d_6-DMSO) δ 170.4, 138.4, 134.8, 128.9, 127.6, 125.2, 121.2, 37.3, 34.7, 24.6. HRMS(ESI) exact mass calcd. for $C_{20}H_{26}B_2N_2O_6S_2$: m/z 477.1499 ([M+H]+); found: m/z 477.1504 ([M+H]+).

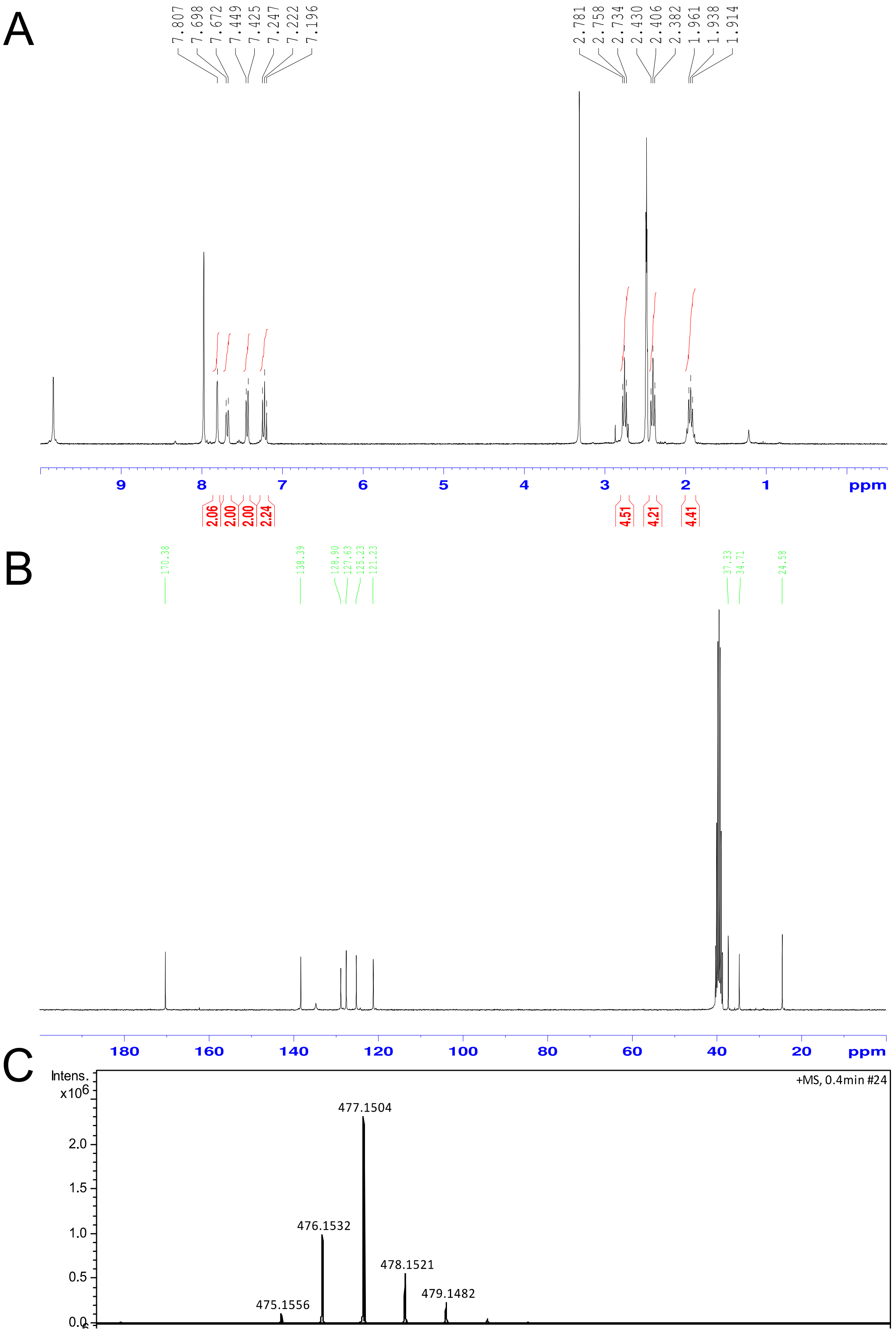

Figure S5. (a) ^{1}H NMR (b) ^{13}C NMR, and (c) ESI+ HRMS spectra of BA-C_5-SH

3-(7-mercapto-2-oxoheptyl)phenylboronic acid (BA-C_7-SH)

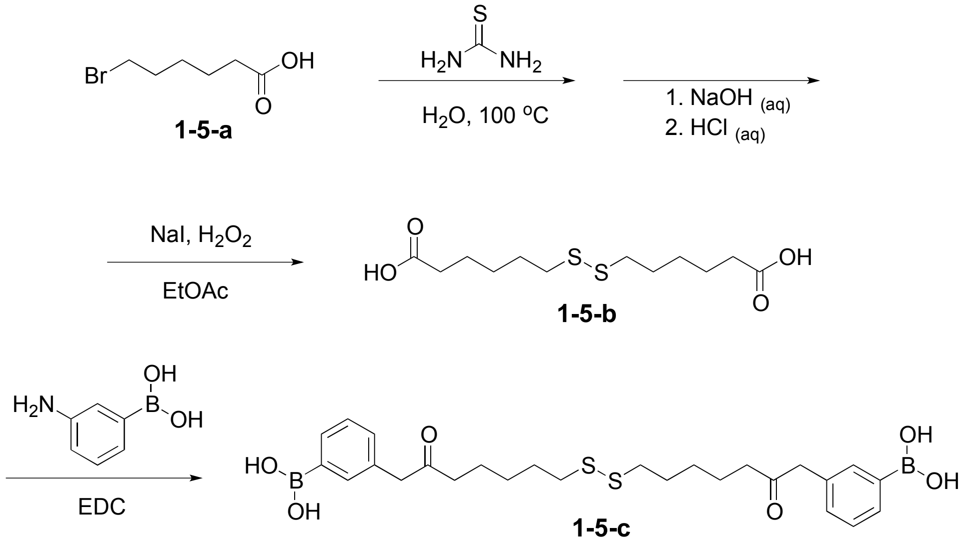

Scheme S5. Scheme of synthesis of 3-(7-mercapto-2-oxoheptyl)phenylboronic acid (BA-C_7-SH)

6-bromohexanoic acid**(1-5-a)** is added into 250mL flask which is contained 30 mL ddH_2O with thiourea (1 eq). The reaction is refluxed and heated up to 100 °C under N_2 atmosphere for 12 hours. Removing the heat, the mixture is cooled down to room temperature and precipitated white crystal. 2M $NaOH_{(aq)}$ is added dropwise into the mixture, stirred for 2 hours at 0 °C. The resulting mixture is dissolved in ether extracted with 1 M $HCl_{(aq)}$ and brine. The combined organic layer is dried over anhydrous $MgSO_4$ and filtered, and solvents are evaporated under reduced pressure. Column chromatography with ether and hexane gives the pure product **(1-5-b)**.

Compound **(1-5-b)** is added to a solution of 100 mL EA with NaI (0.01eq) stirred vigorously for 5 minutes. The mixture is continues stirred for another 30 minutes after adding 1M H_2O_2. Confirming the reaction, mixture is extracted with EA, H_2O and brine. The combined organic layer is dried over anhydrous $MgSO_4$ and filtered, and solvents are evaporated under reduced pressure. Column chromatography with EA and hexane gives the pure product **(1-5-c)**.

Product **(1-5-c)** is added into anhydrous dimethylforamide (10 mL) stirred for 10 minutes. Commercial 3-aminophenylboronic acid (1 eq) is sebsequently

added into the mixture and stirred for another 10 minutes. Coupling reagent, EDC (1.5eq), is added into reactant under dry N_2 atmosphere stirred for 12 hours at 4°C. The reaction can be confirmed by TLC in ninhydrin stain. The resulting mixture is added a small amount of toluene in order to azeotrope with dimethylforamide and evaporated under reduced pressure. Column chromatography(dichloromethane /Methanol, 10:1, v/v) gives the pure product **(1-5-d)**.

^{1}H NMR (300MHz, d_6-DMSO) δ 7.80 (s, 2H), 7.70 (d, J = 7.6 Hz, 2H), 7.44 (d, J = 7.6 Hz, 2H), 7.23 (t, J = 7.6Hz, 2H), 2.70 (t, J = 7.2 Hz, 4H), 2.28(t, J = 7.2 Hz, 4H), 1.68 - 1.56(m, 8H), 1.39 - 1.34(m, 4H). ^{13}C NMR (75.4 MHz, d_6-DMSO) δ 171.0, 138.5, 128.8, 127.6, 125.2, 121.1, 37.7, 36.2, 28.4, 27.5, 24.8. HRMS(ESI) exact mass calcd. for $C_{24}H_{34}B_2N_2O_6S_2$: m/z 533.2126 ([M+H]+); found: m/z 533.2082 ([M+H]+).

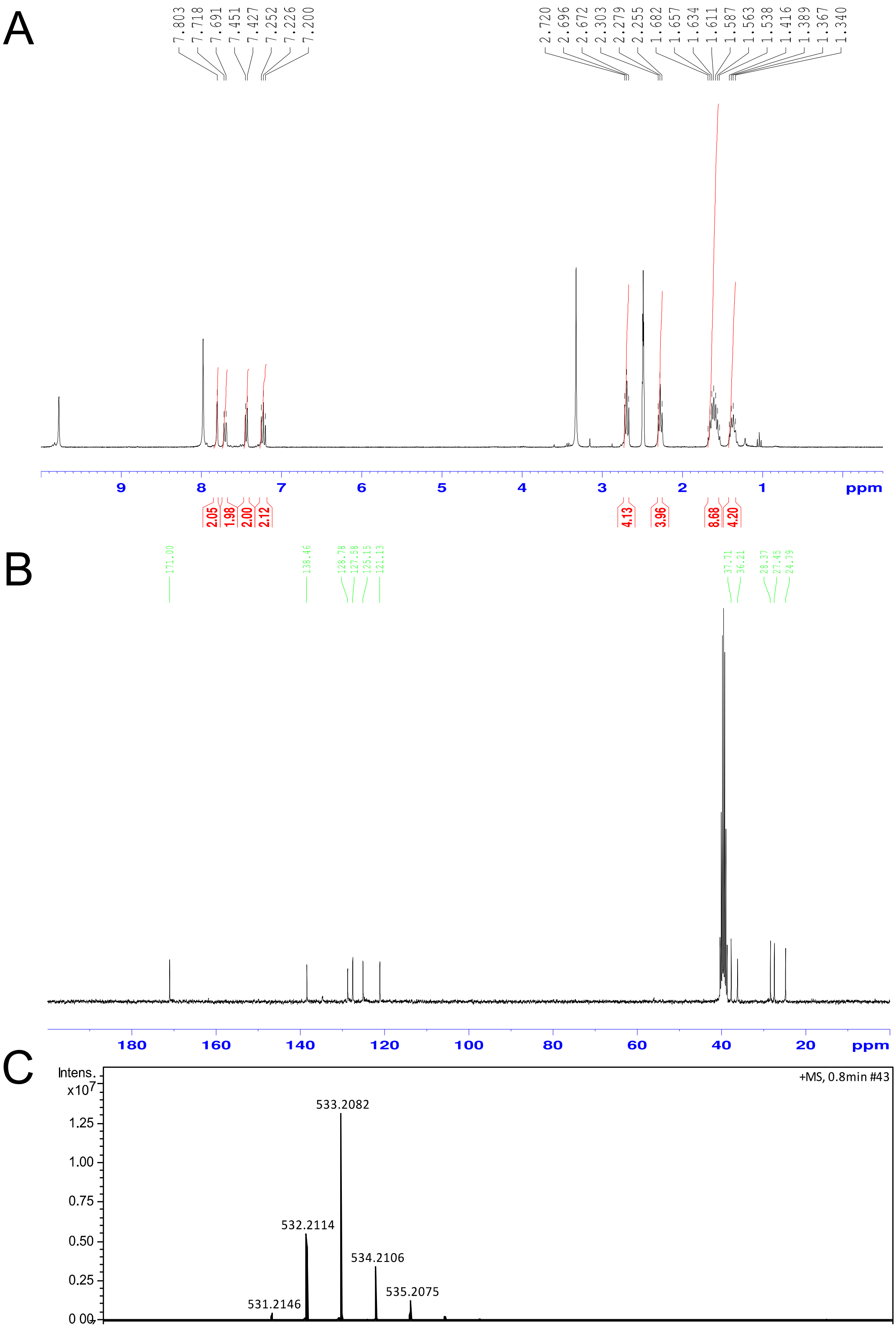

Figure S6. (A) ^{1}H NMR (B) ^{13}C NMR, and (C) ESI+ HRMS spectra of BA-C$_7$-SH
